# Metabolic Perturbations Associated with both PFAS Exposure and Perinatal/Antenatal Depression in Pregnant Individuals: A Meet-in-the-Middle Scoping Review

**DOI:** 10.1007/s40572-024-00451-w

**Published:** 2024-06-19

**Authors:** Himal Suthar, Roselyn B. Tanghal, Lida Chatzi, Jesse A. Goodrich, Rachel Morello-Frosch, Max Aung

**Affiliations:** 1 Department of Population and Public Health Sciences, Keck School of Medicine, University of Southern California, SSB 225R, 1845 N Soto St., Los Angeles, CA 90032 USA; 2 Department of Environmental Science, Policy, and Management, University of California, 130 Mulford Hall #3114, Berkeley, CA 94720 USA

**Keywords:** Per- and poly-fluoroalkyl substances, Metabolic pathways, Mental health

## Abstract

**Purpose of Review:**

Depression during the perinatal or antenatal period affects at least 1 in 10 women worldwide, with long term health implications for the mother and child. Concurrently, there is increasing evidence associating maternal exposure to per- and poly-fluoroalkyl substances (PFAS) to adverse pregnancy outcomes. We reviewed the body of evidence examining both the associations between PFAS exposure and perturbations in the maternal metabolome, and the associations between the maternal metabolome and perinatal/antenatal depression. Through this, we sought to explore existing evidence of the perinatal metabolome as a potential mediation pathway linking PFAS exposure and perinatal/antenatal depression.

**Recent Findings:**

There are few studies examining the metabolomics of PFAS exposure—specifically in pregnant women—and the metabolomics of perinatal/antenatal depression, let alone studies examining both simultaneously. Of the studies reviewed (*N* = 11), the majority were cross sectional, based outside of the US, and conducted on largely homogenous populations. Our review identified 23 metabolic pathways in the perinatal metabolome common to both PFAS exposure and perinatal/antenatal depression.

**Summary:**

Future studies may consider findings from our review to conduct literature-derived hypothesis testing focusing on fatty acid metabolism, alanine metabolism, glutamate metabolism, and tyrosine metabolism when exploring the biochemical mechanisms conferring the risk of perinatal/antenatal depression due to PFAS exposure. We recommend that researchers also utilize heterogenous populations, longitudinal study designs, and mediation approaches to elucidate key pathways linking PFAS exposures to perinatal/antenatal depression.

**Supplementary Information:**

The online version contains supplementary material available at 10.1007/s40572-024-00451-w.

## Introduction

At least 1 in 10 women experience depression during pregnancy or in the postpartum period [1, 2]. Immigrant women and women of color may be at increased risk of perinatal and postpartum depression due to chronic experiences of discrimination and the confluence of other stressors associated with their intersectional identity [3]. Perinatal depression can increase the risk of other adverse maternal health outcomes, including all-cause mortality and suicide [4], cardiovascular conditions [5], and autoimmune disease – although evidence suggests bidirectional relationships [6]. Furthermore, perinatal depression can also impact child health outcomes. For example, a meta-analysis of maternal depression during pregnancy indicated increased risk of adverse pregnancy outcomes such as preterm birth and low birthweight [7]. Additionally, postpartum depression has been associated with poorer mother-to-infant bonding and challenges with breastfeeding [8]. Another meta-analysis of depression during pregnancy and postnatally also found evidence of increased risk for adolescent emotional and behavioral problems and impaired cognition [9]. Thus, understanding potentially modifiable risk factors may aid in characterizing risk of maternal depression and inform prevention efforts.

Per- and poly-fluoroalkyl substances (PFAS) are ubiquitous and persistent organic chemicals that have been widely detected in the environment and in humans. PFAS contamination in environmental media can occur from multiple sources, including waste disposal and atmospheric release from industrial sites, manufacturing sites of PFAS-containing products, use of aqueous film-forming foams, and leaching from landfills and waste sites [10]. The National Academies of Science, Engineering, and Mathematics (NASEM) systematically reviewed human health literature on PFAS and found evidence of risk for metabolic factors such as lipid homeostasis and liver damage, in addition to infant growth early in life, and certain cancer types (breast, kidney and testicular) [11]. Importantly, this NASEM report also underscored major scientific data gaps on neurological health effects associated with PFAS exposure [11]. There are limited observational human studies that have investigated PFAS exposure in association with maternal depression. For example, a study of pregnant women based in San Francisco, CA, identified prenatal PFAS mixtures associated with increased depression scores during pregnancy, particularly among immigrant women, using the Center for Epidemiological Studies-Depression (CES-D) instrument [12•]. However, a separate study of pregnant women based in Cincinnati, OH, did not identify notable associations between PFAS exposures and perinatal and postpartum depression when using the Beck Depression Inventory (BDI-II) instrument [13], despite this study reporting higher levels of all PFAS measured in participants compared to the participants in the San Francisco study mentioned above. Another study in the National Health and Nutrition Examination Survey focused on both male and female adults and found non-linear relationships with depression, such that participants with total PFAS concentrations (sum of perfluorooctanoic acid (PFOA), perfluorooctane sulfonic acid (PFOS), 2-(N-methyl-perfluorooctane sulfonamido) acetate (MPAH), perfluorodecanoic acid (PFDE), perfluoroundecanoic acid (PFUA), perfluorohexanesulphonic acid (PFHxS), and perfluorononanoic acid (PFNA)) above a threshold of 39.6 ng/mL had higher odds of depression using the Patient Health Questionnaire (PHQ-9) instrument [14•]. While findings have been mixed in observational studies, increasing evidence from experimental studies indicates that exposures to PFAS poses a risk for neurobehavioral outcomes, including depression [15•].

The underlying biological mechanisms driving depression are multifaceted. Cao and Ng (2021) reviewed multiple experimental studies and proposed that PFAS may induce neurotoxic effects potentially through promoting peripheral and neuroinflammation and disrupting calcium ion and neurotransmitter signaling [15•]. Further, many studies have shown that PFAS are associated with altered molecular signatures across multiple metabolic pathways relevant for inflammation, including fatty acid biosynthesis and amino acid metabolism [16••]. Application of metabolomics assays in human observational studies may provide insight into biological pathways linking PFAS exposure to perinatal/antenatal depression outcomes. Metabolomics is the systematic measurement of metabolites in the human body. Recent technological advancements have allowed metabolomics assays to quantify the levels of hundreds or thousands of biologically active small molecules in a variety of different biospecimens. The three primary methods for measuring metabolomics are liquid chromatography-mass spectroscopy (LC–MS) [17], gas chromatography-mass spectroscopy (GC–MS) [18], and nuclear magnetic resonance spectroscopy (NMR) [19]. Depending on the technology employed, metabolomics can measure a broad range of organic compounds, such as amino acids, lipids, fatty acids, nucleotides, steroids, and flavonoids, among others. Since changes in metabolites reflect alterations in metabolic pathways that can be related to both environmental factors and diseases, obtaining a comprehensive view of these different types of metabolites can provide insight into the biological mechanisms of environment associated disease. Increased accessibility and developments in software performing pathway analysis – the algorithmic grouping of metabolites into larger functional groups – have also helped researchers contextualize perturbations in individual metabolites within the broader scope of the biological systems they are involved in [20]. Non-targeted metabolomics is particularly useful in understanding perturbations in metabolic pathways, because it measures a broad range of metabolic classes.

Previous studies have separately evaluated metabolomics in the context of PFAS exposure and maternal depression [16••, 21••]. Therefore, we aim to utilize this body of evidence to qualitatively assess priority in metabolic pathways that are common between PFAS exposure and perinatal/antenatal depression. In this review, we aim to 1) summarize the characteristics, methodologies, and findings of studies in this field; 2) identify potential metabolic pathways that may be considered as mediators in the biological pathway between PFAS exposure and perinatal/antenatal depression; and 3) provide recommendations for future studies.

## Review Methods

For this review we utilized a meet-in-the-middle-approach to identify metabolic pathways associated with both PFAS exposure in pregnant women and perinatal/antenatal depression. The review consisted of a literature search, article selection based on title and abstract review, article refinement based on full text review, data extraction, and data synthesis.

### Literature Search

Two literature searches were conducted in compliance with the PRISMA methodology [22] for scoping reviews on PubMed, PMC, and MedLine databases between December 2023 and February 2024: one for primary data articles investigating the metabolics pathways associated with PFAS exposure in pregnant women using untargeted metabolomics, and one for primary data articles investigating the associations between metabolic pathways and perinatal/antenatal depression in women using untargeted metabolomics. The exact search terms utilized for conducting the literature searches can be found in Supplemental Table 1 and Supplemental Table 2.

### Article Selection and Exclusion Criteria

Two researchers independently conducted both literature searches and screened the titles and abstracts for salient articles. Articles common to both researchers’ screenings were selected for full text review; discrepancies between the researchers’ selections were discussed and moved forward after approval by the corresponding author. We included all relevant articles excluding those which were 1) animal studies; 2) review articles; 3) studies conducting targeted metabolomics approaches; 4) studies that did not perform pathway analysis using associated metabolites and 5) studies that did not analyze biospecimen sampled from women in the perinatal or postpartum periods. Targeted metabolomics was an exclusion criterion in our review to allow for broader comparisons of significant metabolic pathways across studies, as studies utilizing targeted metabolomics would need to measure identical metabolomic assays to be compared. Studies which did not perform pathway analysis were excluded as pathway analysis allows for the standardization of both metabolites and their larger functional pathways, facilitating more generalizable comparisons at a systems biology level rather than at an individual metabolite level, which is more susceptible to interstudy variability.

### Data Extraction

Once the articles to be included in this scoping review were finalized, key characteristics of each article were extracted.

For articles investigating the associations between PFAS exposure and metabolomics in pregnant women, we extracted and recorded information from each study using a pre-specified data extraction form including information on description of population, sample size, whether the sample was diverse with regards to ethnic or sociodemographic composition, PFAS compounds measured, biospecimen used for PFAS exposure assessment, PFAS analytical measurement method, biospecimen used for metabolic pathway analysis, metabolomic profiling method, tool used to conduct metabolic pathway analysis, significant metabolic pathways associated with PFAS exposure, and whether PFAS mixtures analysis was conducted.

For articles investigating the metabolomic profiles of women with perinatal/antenatal depression, we extracted and recorded information from each study using a pre-specified data extraction form including information on: description of population, sample size, whether the sample was diverse with regards to ethnic or sociodemographic composition, study design, biospecimen used for metabolic pathway analysis, metabolomic profiling method, tool used to conduct metabolic pathway analysis, depression outcomes measured, depression outcome measurement method, and significant metabolic pathways associated with perinatal/antenatal depression.

Within each article, metabolic pathways associated with either PFAS exposure or perinatal/antenatal depression were identified and standardized according to Supplemental Table 3 to allow for better harmonization of pathways derived from the different technologies used for metabolomics and tools used to conduct pathway analysis across the studies. For instance, pathways which were combinations of multiple sub-pathways were differentiated into their component parts (e.g., “Alanine and Aspartate metabolism” would be separated into “Alanine metabolism” and “Aspartate metabolism”). The standardized pathways were then ranked in descending order based on author-provided p-values, effect sizes, number of significant signatures, or a combination of the three. To more thoroughly identify all potential pathways, pathways were selected based on p-values (if provided) rather than q-values, and, if a study conducted a mixtures analysis, then pathways significant across both pairwise and mixtures analyses were combined. For pathways which were combinations of multiple sub-pathways, the same rank was assigned to each of the sub-pathways. For example, if “Alanine and Aspartate metabolism” was ranked as the second most significant pathway in a given study, then both “Alanine metabolism” and “Aspartate metabolism” would be given the rank of 2. If a pathway’s standardized form appeared multiple times, then the first instance (i.e., the most significant ranking) was retained and ranked, and all other instances deleted.

All steps in the extraction process were performed individually by two researchers, with discrepancies reconciled through additional review and discussion facilitated by the corresponding author.

### Data Synthesis

Following data extraction, data synthesis was performed to identify metabolic pathways in pregnant women associated with both PFAS exposure and antenatal/perinatal depression. First, all significant standardized pathways from each study across both review arms were amalgamated. Through manual review, standardized pathways common to both arms of the review were identified. Finally, for each article in each arm, the common pathways were ranked in descending order of significance, removing standardized pathways which were not common from the rankings. For example, if an article identified Glycine metabolism, BCAA metabolism, and TCA Cycle as significant pathways (in descending order), and BCAA metabolism was not a pathway common to both arms of the review, then the common pathway ranking for that article would be Glycine metabolism (rank order 1), TCA Cycle (rank order 2).

### Assessment

Potential mediation pathways were prioritized based on significance rank within studies and significance across studies. Further, qualitative commentary summarizing the current state of research in this field was developed based on key characteristics of the final articles reviewed.

## Results

**Fig. 1:**
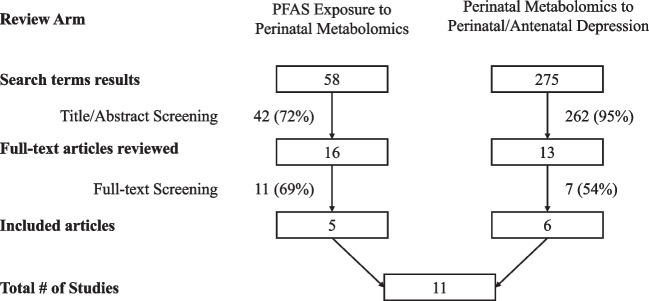
Funnel diagram depicting study selection process

The literature search retrieved 333 unique articles (58 pertaining to PFAS exposure and perinatal metabolomics; 275 pertaining to metabolomics and perinatal/antenatal depression; Fig. 1). Of these, 16 articles were selected for full text review based on title and abstract screening in the PFAS exposure to perinatal metabolomics arm, and 13 articles were selected for full text review in the metabolomics to perinatal/antenatal depression arm. Based on full text review, five articles were selected to undergo data extraction in the review arm focusing on the associations between PFAS exposure and perinatal metabolomics, and six articles in the review arm focusing on the metabolomics of perinatal/antenatal depression.

### Summary of Studies Identified for PFAS exposure to Perinatal Metabolomics

A total of five articles studying the perinatal metabolomics of PFAS exposure were included in this review (Fig. 1) [23•, 24•, 25, 26, 27•], and the key characteristics of these papers are listed in Supplemental Table 4. All studies measured PFAS exposure utilizing mass spectroscopy on maternal blood, with four utilizing serum as the biospecimen and one utilizing plasma. The study sample sizes varied from 84 to 459. Of these five studies, four utilized a cross-sectional design and one was a nested case–control study investigating breast cancer as the primary outcome. A combination of legacy and novel PFAS were measured in all articles in this review arm. The lowest number of unique PFAS compounds measured was four [24•, 27•] and the highest number of PFAS measured was 11 [26]. All studies measured PFOS, PFOA, PFNA, and PFHxS, while one study included perfluorodecanoic acid (PFDA) and PFUA in addition to the four types of PFAS mentioned above. Two papers measured more than seven types of PFAS [25, 26]. For both measuring PFAS and metabolomics, the earliest biospecimen was taken between 6 to 17 weeks’ gestation [27•], and the latest biospecimen at early postpartum 1 to 3 days after delivery [26]. Three studies performed pathway analysis using Mummichog [28], one with MetaboAnalyst (available at https://www.metaboanalyst.ca/MetaboAnalyst/home.xhtml), and one employing a 3rd party. One study population originated from China [25] and the remaining studies sampled populations based in the United States. Of those four studies, two studies focused on pregnant African American women in Atlanta, Georgia [24•, 27•], and two studies [23•, 26] collected data from pregnant women who identified as Black, White, or Hispanic. Only two studies [23•, 27•] conducted a mixtures analysis to measure the effect of the PFAS mixture on the perinatal metabolomic profile, and both utilized the quantile g-computation methodology [29].

### Summary of Studies Identified for Metabolomics to Perinatal/Antenatal Depression

A total of six articles studying the metabolomics of perinatal/antenatal depression were included in this review (Fig. 1) [30•, 31•, 32•, 33, 34•, 35]. The key characteristics of these studies are summarized in Supplemental Table 5. Four studies performed untargeted metabolomics on blood samples, with three using serum [30•, 31•, 33] and one using plasma [34•]. One study utilized urine samples [35] and one utilized cerebrospinal fluid [32•]. Sample sizes of the studies ranged from 20 to 431, with three studies having a sample size below 100. Three studies leveraged a cross-sectional study design, two a cohort study design, and one a longitudinal design. Three studies utilized gas chromatography-mass spectroscopy, one study utilized liquid chromatography-tandem mass spectrometry, and two studies utilized nuclear magnetic resonance spectroscopy/plasma-mass spectrometry. Metabolomics were performed on specimens collected between 8 weeks gestation and 5 weeks postpartum. Four studies collected specimen for metabolomics during the perinatal period, one collected specimen during the postpartum period, and one study collected specimens during both the perinatal and postpartum periods. Four studies utilized MetaboAnalyst for pathway analysis, one utilized the KEGG Pathway Database, and one utilized the Kyoto Encyclopedia of Genes and Genomes. Four studies measured postpartum depression as an outcome and two measured perinatal depression or depression likeliness. Five of the six studies determined depression or depression likeliness using the Edinburgh Postnatal Depression Scale (EPDS) [36], and one study used the Diagnostic and Statistical Manual of Mental Disorders, 4th edition (DSM-IV) [37]. The study populations of all six studies were from outside the United States, with four based in China, one in Canada, and one in Japan.

### Common Pathways Identified

**Fig. 2:**
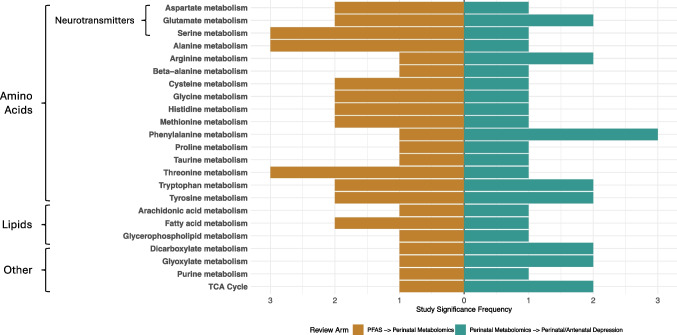
Significance frequency of common metabolic pathways by review arm. Metabolic pathways identified by both Chang et al., 2022 [24•] and Liang et al., 2023 [27•] in the perinatal metabolomics of PFAS exposure review arm were only counted once between the two studies due to a high degree of overlap in sample population and measurement methods

The common pathways identified in both arms of the review as well as their occurrence frequencies across studies are shown in Fig. 2. Pathways identified by both Chang et al., 2022 [24•] and Liang et al., 2023 [27•] were only counted once in Fig. 2 due to similar sample populations and measurement methods; absolute occurence frequencies are shown in Table 1. A total of 23 metabolic pathways were identified which were associated with both perinatal PFAS exposure and perinatal/antenatal depression. Of the common pathways, alanine metabolism was the pathway with the most signatures (*n* = 4) in studies focusing on the metabolomic profiles of perinatal PFAS exposure, and phenylalanine metabolism was the most common significant pathway in studies investigating metabolomics of perinatal/antenatal depression (*n* = 3, Table 1). Alanine metabolism (*n* = 5), glutamate metabolism (*n* = 5), and tyrosine metabolism (*n* = 5) were the most common metabolic pathways found in studies across both review arms. Finally, the fatty acid metabolism pathway was found to be the pathway with the highest significance most often within studies (*n* = 3).

**Table 1:** Significance frequencies and ranking of common metabolic pathways in studies across review arms. “X” denotes if a metabolic pathway was found significant within a study; “*” denotes if a metabolic pathway was the most significant pathway found within a study

				Review Arm										
				PFAS Exposure to Perinatal Metabolomics					Metabolomics of Perinatal/Antenatal Depression					
			Study	Chang, 2022	Hu, 2020	Li, 2021	Liang, 2023	Prince, 2023	Laketic, 2023	Lin, 2017	Mao, 2021	Sheng, 2023	Yang, 2024	Yu, 2022
			Biospecimen	Serum	Serum	Serum	Serum	Plasma	Serum	Urine	Serum	Cerebrospinal Fluid	Serum	Plasma
Major Group	Sub-Group	Common Pathways	Article Significance Frequency											
Amino Acids	Neurotransmitters	Aspartate metabolism	3	X				X			X			
		Glutamate metabolism	5	X			X	X			X		*	
		Serine metabolism	4		X		X	X			*			
		Alanine metabolism	5	X	X		X	X			X			
		Arginine metabolism	4	*			X					X	X	
		Beta-alanine metabolism	2	X								X		
		Cysteine metabolism	3	X				X	X					
		Glycine metabolism	3				X	X			*			
		Histidine metabolism	4	X			X	X					X	
		Methionine metabolism	3	X				X	X					
		Phenylalanine metabolism	4			X			*	X		X		
		Proline metabolism	2	*									X	
		Taurine metabolism	2					X		*				
		Threonine metabolism	4		X		X	X			*			
		Tryptophan metabolism	4	X				X	X			X		
		Tyrosine metabolism	5	X			X	X	X			X		
Lipids		Arachidonic acid metabolism	2			*					X			
		Fatty acid metabolism	4	X			*	*				*		
		Glycerophospholipid metabolism	3	X			X				X			
Other		Dicarboxylate metabolism	3				X			X	X			
		Glyoxylate metabolism	3				X			X	X			
		Purine metabolism	2	X								X		
		TCA Cycle	3				X			X				*

## Discussion

Multiple studies have suggested a possible link between PFAS and poorer mental health outcomes [12•, 14•]. Concurrently, metabolomics are increasingly being leveraged to identify biochemical profiles of physiological states, and show promise in disentangling the relationship between PFAS and mental health during and immediately after pregnancy. To the best of our knowledge, this is the first review utilizing a two-pronged approach to identify potential metabolic pathways linking PFAS exposure with perinatal and/or antenatal depression. We identified several candidate pathways, including fatty acid metabolism and amino acid metabolism which may mediate this relationship. In addition, this first of its kind review approach to identify potential mechanisms linking environmental factors with disease can serve as a template for future researchers to apply to other exposures and outcomes.

### Fatty Acid Metabolism

In our meet-in-the-middle review, fatty acid metabolism was highlighted as a significant pathway associated with PFAS exposure and perinatal/antenatal depression. Fatty acids make up important components of lipids in the cellular membrane and are major sources of energy [38]. PFAS has been linked with the dysregulation of fatty acid metabolism in the metabolomic profile of adults [39]. Fatty acid metabolism was also associated with depression during the postpartum period, with a study highlighting that higher levels of polyunsaturated fatty acids were associated with greater odds of depression [40]. Arachidonic acid, for example, is a derivative of polyunsaturated fatty acid and has been shown to play a significant role in the physiological functions of every life stage [41], and linked with inflammation and neurological disorders [42].

Disruptions in the fatty acid pathway can result in changes to the metabolic programming which have the potential to increase risk of metabolic diseases in later life [43]. Exposure to PFOA, a specific type of PFAS, was found to be associated with an increase of fatty acid oxidation in young adults, potentially hindering glucose metabolism [44]. Disruption in arachidonic acid metabolism has been linked with inflammation, and some secondary eicosanoid metabolites are associated with neurodevelopment [45]. Neuroinflammation has been hypothesized to be a main factor for the onset of major depression disorder (MDD) and there are studies that show depressed adults having elevated levels of inflammatory factors [46–48]. Future studies should continue to investigate arachidonic and fatty acid metabolism as a precursor to adverse child health outcomes.

### Amino Acid Metabolism

Amino acids, a major sub-group of the human metabolome, are the base components of proteins and key signaling compounds including neurotransmitters and hormones [49, 50]. Our meet-in-the-middle review revealed that metabolism of three amino acids – alanine, glutamate, and tyrosine – were the most frequently identified significant metabolic pathways across both review arms. Previous studies have shown PFAS exposure to be associated with alterations in amino acid metabolism [51], with multiple studies consistently identifying tyrosine metabolism and alanine metabolism as pathways perturbed by PFAS exposure [51–53]. Alanine and glutamate are non-essential amino acids which have been shown to have high positive correlations with HAM-D depressive scores [54], with one study identifying alanine as a significant discriminator of suicidal attempt among those with MDD [55]. Cerebrospinal fluid levels of tyrosine, a conditionally essential amino acid, have also been implicated with MDD [56].

These amino acids are pivotal beyond pregnancy, as amino acid metabolism has been shown to play a vital role in physical growth and neurocognitive development during early life [57]. Specifically in the context of neurobehavior, all three of the amino acids identified in our review have been shown to be signatures of both the presence and severity of autism in children [58]. Lower levels of alanine and tyrosine were found in children with autism compared to controls [58, 59], while glutamate, a major excitatory neurotransmitter [60], was shown to be associated with improved socialization in boys with autism [61]. Due to an increasing body of evidence suggesting that the infant metabolome largely reflects the maternal metabolome [62], perturbations in the maternal metabolome as a result of environmental exposures are particularly useful for further study of the environmental impact on child outcomes.

### Amino Acids as Neurotransmitters

Glutamate, aspartate, and glycine are amino acids that act as mediators in the transmission of nerve impulses [63, 64], all three of which were identified as common metabolic pathway signatures of PFAS exposure and perinatal/antenatal depression. Glutamate is an abundant neurotransmitter in the brain, and receptors of glutamate are involved in developmental processes including learning, memory formation, and synaptic plasticity [65]. As mentioned above, glutamate is a potential biological marker of autism spectrum disorder (ASD) as findings have shown that individuals with ASD have elevated levels of glutamate in their brains, and these levels were indicative of increased gliosis interrupting enzyme regulation and metabolism of glutamate [66]. Further, glycine is an inhibitory neurotransmitter found in the spinal cord and assists in processing auditory information [67]. Similar to glycine, aspartate is found in the spinal cord, however, aspartate is an excitatory neurotransmitter that increases the likelihood of depolarization during of the postsynaptic membrane [63].

### Strengths and Limitations

Our review has notable strengths. First, is in its unique meet-in-the-middle approach to exploring potential metabolic mediators linking prenatal and perinatal PFAS exposure to perinatal/antenatal depression. Second, is the thoroughness of the data extraction and synthesis performed across studies reviewed, as two researchers independently assessed the significant pathways based on commonality, inter-study significance frequency, and intra-study significance ranking, bolstering confidence in the internal validity of our findings. Third, is the specificity of our study selection with regards to the sample population and time at which key outcomes and exposures were evaluated, as our review only included studies which measured the PFAS exposure, metabolomic profile, and presence of depression in women during the perinatal or antenatal period. This targeted approach allows us to more precisely draw inferences on the temporal nature of these associations in this specific population.

There are also limitations of this review to consider. Our review is limited to studies that conduct metabolic pathway analysis; therefore, we were unable to assess the relationship of individual metabolites with PFAS exposure and perinatal/antenatal depression, or evaluate pathways implied by significant individual metabolites which were not identified through a formal pathway analysis. This approach also did not allow us to evaluate the direction of associations between metabolic pathways and PFAS exposure and perinatal/antenatal depression, as pathway analysis summarizes metabolites with significant associations without producing effect estimates. Further, the heterogeneity in biospecimen analyzed across the studies limits our ability to generalize our findings to the entire human physiology, as the metabolic profiles vary by factors including fasting status, tissue sampled for analysis, and diet [68, 69]. Finally, the variation in tools leveraged to perform pathway analysis across the studies confers risk to the validity of our intra-study findings. While this is partially ameliorated by our standardization process, there is still the possibility of heterogeneity in classifying metabolites dependent on analysis tool.

## Conclusion

This scoping review article presents an approach for building the evidence base to investigate metabolic pathways that may link PFAS exposures and perinatal/antenatal depression. Based on the extracted literature, we propose the following recommendations and considerations to build on our review: selection of a more socioeconomically/ethnically diverse sample; leveraging mixtures analysis to more accurately measure the effects of PFAS exposure as they occur naturally; utilizing more longitudinal (i.e., cohort) based study designs to study the temporal effects of PFAS exposure on the perinatal metabolome and maternal depression; advancing evidence for establishing biomarker fingerprints of both PFAS exposure and perinatal/antenatal depression; and, specifically investigating the roles of amino acid metabolism and fatty acid metabolism, and their respective metabolites, as potential mediators conferring the risk of PFAS exposure on perinatal/antenatal depression leveraging existing frameworks of multivariate mediation analysis of environmental data [70].

### Supplementary Information

Below is the link to the electronic supplementary material.

Supplementary file1 (XLSX 26.4 KB)

## Data Availability

No datasets were generated or analyzed for the current review. All included and excluded studies based on full text review can be found in our Supplementary Material for readers to evaluate with review criteria.
